# Application of the New Process for Unapproved Drug Use: Dilemma of Universal Health Care Coverage in Japan

**DOI:** 10.1200/JGO.19.00313

**Published:** 2019-11-08

**Authors:** Satoshi Nishiwaki, Yuichi Ando

**Affiliations:** Satoshi Nishiwaki, MD, PhD and Yuichi Ando, MD, Nagoya University Hospital, Nagoya, Japan

## TO THE EDITOR:

Patient-proposed health services (PPHS; *kanjya mouside ryouyou* in Japanese), a new Japanese government program that allows patients to use publicly insured and uninsured therapies—a so-called mixed medical care service that is prohibited in principal—was introduced in April 2016.^1^ Under PPHS, a patient can petition the Ministry of Health, Labour, and Welfare (MHLW) to assess an unapproved treatment by submitting documents from a Medical Service Act–certificated core clinical research hospital, where a study protocol is prepared after a review of the proposed treatment from the following points of view: (1) no possible participation in the existing system, such as ongoing clinical trials or Advanced Medical Care B^2^; (2) the safety and efficacy of the treatment; and (3) the possibility of future approval of the treatment. The distinctive feature of this system is that the required care is performed as clinical research to accelerate the approval of the treatment. PPHS is a unique system designed to support patient wishes regarding the earliest possible advanced medical care without disrupting Japan’s universal health care coverage.^3^

We experienced the first case in which PPHS was associated with approval of thiotepa. Thiotepa is an alkylating agent approved in 1958 as Tespamin (Osaka, Japan) in Japan. However, production in Japan ended in 2009 due to circumstances related to the pharmaceutical company marketing the drug. In July 2016, we received a proposal to use thiotepa as a conditioning drug for autologous stem-cell transplantation under PPHS. At approximately the same time, in November 2016, a clinical trial of thiotepa (DSP-1958) was launched by Sumitomo Dainippon Pharma (Osaka, Japan). However, the patient being considered for PPHS use did not meet the criteria for the clinical trial. To minimize the patient cost burden, Adienne Pharma & Biotech (Lugano, Switzerland) offered to provide Tepadina (their marketed brand of thiotepa), which had been approved by the European Medicines Agency in 2010, free of charge. The PPHS application was approved in May 2017 by the MHLW, and the patient underwent autologous stem-cell transplantation in early June 2017, using thiotepa as a part of the conditioning regimen. Subsequently, in March 2019, DSP-1958 was approved in Japan. This patient case shows that PPHS can serve as a last-resort bridge to approval.

With regard to this patient case, the distinction between PPHS and the Compassionate Use Program (CUP)^1^ is related to two factors: timing and rareness of the disease. A prerequisite for a CUP is that the medication use will not adversely affect the implementation of the main clinical trial. Therefore, in principle, it is possible to implement a CUP after completion of the main clinical trial. However, including the preparation time for a CUP protocol, it is impossible to use the drug, either in the main clinical trial or in a CUP, for at least several months. This patient case showed that PPHS could fill in this gap by acquiring accurate information about the main clinical trial and the CUP and preparing as quickly as possible.

The Japanese government positioned PPHS as a part of the Japan Revitalization Strategy, as revised in 2014.^4^ There appears to be a gap in the concept of PPHS between the patient’s hope and reality. First, PPHS is designed on the premise of maintaining universal health care coverage in Japan. Patients find it difficult to accept that it takes at least several months for the core clinical research hospital to prepare the required PPHS documentation. The economic burden on the patient is another problem. Although, in our experience, thiotepa was offered free of charge by the pharmaceutical company, the patient was charged approximately $6,500 to cover clinical trial preparation and implementation costs. This cost was quite low for clinical research, but it was a heavy burden for the patient. As an indication of the various difficulties of PPHS, only seven studies have been implemented under the program through March 2019 (2.3 studies/year).

It remains important to discuss how to handle medical treatments that are not covered by public insurance in Japan, including whether to allow a private insurance–led health care system such as that in the United States.^5^ Alternatively, PPHS might develop into a system similar to the single-patient investigational new drug application (differences are summarized in Table 1).^6,7^ In the short term, it is good to have a mechanism that responds to the requests of individuals; however, from the point of view of drug approval, in the long term, except in limited cases, it may be more generally beneficial for patients to follow the established approval process while at the same time develop policies that would motivate more extensive drug development by Japanese pharmaceutical companies. As they say, “haste makes waste” (*isogaba maware* in Japanese).

**TABLE 1:**
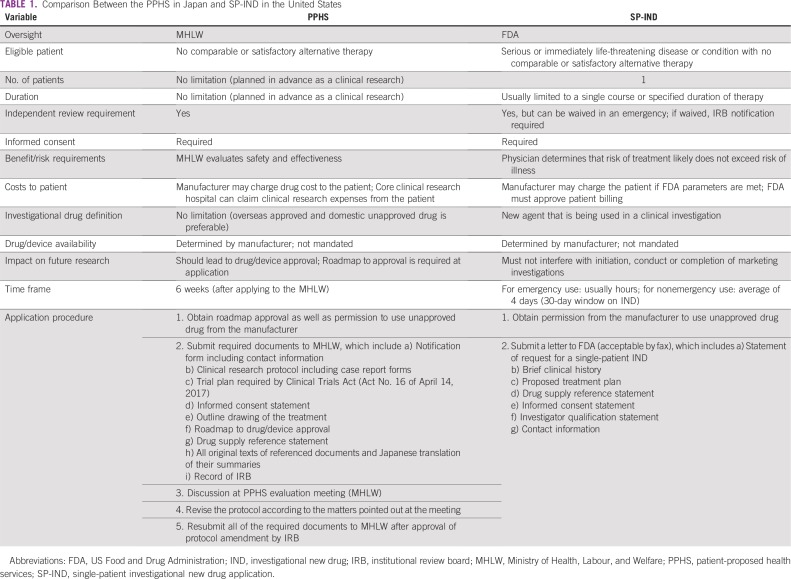
Comparison Between the PPHS in Japan and SP-IND in the United States
